# Photoprotective Effects of Quercetin and Hesperidin in Polymorphous Light Eruption: A Comparative Study with Alpha-Glucosylrutin

**DOI:** 10.3390/cimb47070567

**Published:** 2025-07-19

**Authors:** Yoon-Seo Choi, Sang-Hoon Park, Inhee Jung, Eun-Ju Park, Wonki Hong, Jin-Hee Shin, Won-Sang Seo, Jongsung Lee

**Affiliations:** 1 Graduate School-Interdisciplinary Program in Biocosmetics, Sungkyunkwan University, Suwon 16419, Republic of Korea; eveelf@g.skku.edu; 2 Department of Plastic Surgery, ID Hospital, Seoul 06039, Republic of Korea; spark@idhospital.com; 3 Global Medical Research Center, Seoul 06526, Republic of Korea; ihjung@gmrc.co.kr; 4 Central Research Institute, Daebong LS Co., Ltd., Incheon 21697, Republic of Korea; ej.park@daebongls.co.kr; 5 Central Research Institute, UCL Co., Ltd., Incheon 21697, Republic of Korea; wk.hong@e-ucl.co.kr; 6 P&K Skin Research Center, Seoul 07236, Republic of Korea; jh.s@pnkskin.com; 7 Strategic Marketing Division, UCL Co., Ltd., Seoul 07236, Republic of Korea; 8 Molecular Dermatology Laboratory, Department of Integrative Biotechnology, College of Biotechnology and Bioengineering, Sungkyunkwan University, Suwon 16419, Republic of Korea

**Keywords:** polymorphous light eruption (PLE), quercetin, hesperidin, alpha-glucosylrutin, sun allergy

## Abstract

Polymorphous Light Eruption (PLE) is a prevalent UV-induced photodermatosis characterized by abnormal immune responses, oxidative stress, and cutaneous inflammation. Alpha-glucosylrutin (AGR), a chemically modified flavonoid widely used for its antioxidant and photoprotective effects, has shown clinical efficacy; however, its synthetic origin and classification as a potential skin sensitizer and aquatic toxin raise safety and environmental concerns. These limitations underscore the need for safer, naturally derived alternatives. In this study, we investigated the comparative efficacy of quercetin (QC) and hesperidin (HPN)—two plant-based flavonoids—against AGR in in vitro and ex vivo models of sun-induced skin damage. An optimized QC:HPN 8:1 (*w*/*w*) complex significantly restored antioxidant enzyme activities (SOD: 4.11 ± 0.32 mU/mg; CAT: 1.88 ± 0.04 mU/mg) and suppressed inflammatory cytokine production (IL-6: 155.95 ± 3.17 pg/mL; TNF-α: 62.34 ± 0.72 pg/mL) more effectively than AGR. β-hexosaminidase secretion, a marker of allergic response, was reduced to 99.02 ± 1.45% with QC:HPN 8:1, compared to 121.33 ± 1.15% with AGR. QC alone exhibited dose-dependent cytotoxicity at ≥10 μg/mL, whereas HPN maintained >94% cell viability at all tested concentrations. These findings highlight the QC:HPN 8:1 complex as a safe, natural, and effective alternative to synthetic AGR for preventing and managing PLE and UV-induced dermal inflammation. Further research should focus on clinical validation and formulation development for topical use.

## 1. Introduction

Sun allergy, or photosensitivity, encompasses a range of abnormal skin reactions to ultraviolet (UV) radiation, including rashes, blisters, and severe sunburns, which can substantially impair quality of life [1]. Although conventional photoprotection methods such as sunscreens and physical barriers provide a degree of protection, they are often insufficient to fully prevent UV-induced skin damage, especially in individuals with heightened photosensitivity [2]. This limitation has spurred significant interest in natural compounds with intrinsic photoprotective properties as adjunct or alternative approaches, particularly for managing conditions like Polymorphous Light Eruption (PLE) [3].

PLE is among the most common photodermatoses, affecting approximately 10–20% of individuals in Western populations and up to 15% globally, with a higher prevalence among young women in temperate climates [4,5,6,7]. The condition is primarily triggered by UVA radiation, which is implicated in up to 90% of cases [4,5,8]. Clinically, PLE presents as intensely pruritic, polymorphic skin lesions on sun-exposed areas, often emerging during spring or early summer [4,5,6]. Unlike typical sunburn responses, PLE reflects an aberrant immune reaction to UV exposure characterized by a failure of normal UV-induced immunosuppression and the subsequent development of a delayed-type hypersensitivity response [9,10,11]. PLE patients exhibit a unique immunological profile marked by resistance to the immunosuppressive effects of UV radiation, leading to exaggerated inflammatory responses to photoinduced antigens [9,10,11].

The pathogenesis of PLE is strongly driven by UVA-induced oxidative stress, which initiates a cascade of inflammatory events. UVA exposure promotes the generation of reactive oxygen species (ROS) and lipid peroxidation, which in turn upregulate intercellular adhesion molecule-1 (ICAM-1) expression via activator protein-2 (AP-2) activation in keratinocytes. This process enhances leukocyte adhesion and sustains inflammation in sun-exposed skin [12]. Patients with PLE demonstrate increased oxidative sensitivity, as evidenced by UVA-induced elevation of pro-inflammatory cytokines such as interleukin-6 (IL-6), interleukin-8 (IL-8), and chemokine (C-C motif) ligand 20 (CCL20) in skin models [12].

Photoaging, another significant consequence of chronic UV exposure, shares oxidative stress as a central pathogenic driver but diverges in immune regulation. Photoaging manifests clinically as wrinkles, dyspigmentation, telangiectasia, and xerosis [13,14,15,16], primarily driven by UV-induced upregulation of matrix metalloproteinases (MMPs) such as MMP-1, MMP-3, and MMP-9, which degrade collagen and elastin in the dermal extracellular matrix [16,17]. Concurrently, UV radiation suppresses collagen biosynthesis in fibroblasts through ROS-mediated inhibition of procollagen gene expression [16,18], leading to dermal thinning and compromised skin barrier function [17,19]. While PLE is characterized by acute immune hypersensitivity with preserved immune surveillance [20,21], photoaged skin exhibits chronic immunosuppression, including PD-L1 upregulation and regulatory T cell (Treg) expansion, which facilitate persistent matrix degradation and increase the risk of carcinogenesis [22,23].

The shared oxidative cascade in PLE and photoaging involves ROS-induced DNA, lipid, and protein damage, activation of inflammatory signaling pathways such as MAPK and NF-κB, and upregulation of MMPs that contribute to extracellular matrix breakdown [24,25]. However, the immune responses in these conditions diverge: in PLE, impaired UV-induced immunosuppression leads to excessive inflammation characterized by elevated IL-1β and reduced IL-1 receptor antagonist (IL-1Ra) ratios in UV-exposed skin [20], promoting neutrophil infiltration and delayed-type hypersensitivity reactions. In contrast, photoaging is marked by chronic immune exhaustion and progressive structural decline due to long-term UV-induced immunosuppression [22,23,26,27].

Current photoprotection strategies, particularly those based on antioxidant interventions, have shown efficacy in mitigating ROS-driven skin damage in both PLE and photoaging [28]. Alpha-glucosylrutin (AGR), a synthetic bioflavonoid derivative, has been extensively studied and clinically validated as an adjunctive therapy in PLE management. When combined with tocopheryl acetate and UVA-protective sunscreens, AGR-containing formulations have been shown to reduce PLE lesion development by 50–60% compared to sunscreen-alone controls [29]. Topical application of 0.25% AGR effectively scavenges UVA-induced ROS, preserves endogenous antioxidants such as glutathione, and prevents ICAM-1 overexpression via AP-2 pathway modulation [12,29,30]. Clinical trials demonstrate that pretreatment with AGR-containing formulations for 3–7 days prior to UV exposure can completely prevent PLE development in most patients during photoprovocation testing [30]. Commercial products like Eucerin^®^ Sun Allergy Protection, which incorporate AGR with broad-spectrum UV filters, have demonstrated reliable efficacy in preventing photodermatoses without causing skin irritation [31]. AGR’s primary mechanisms of action include neutralization of hydroxyl, superoxide, and peroxyl radicals, inhibition of stress-activated protein kinases, and suppression of ICAM-1 overexpression [12,29].

Despite its clinical success, AGR presents several limitations. AGR is not a naturally occurring compound but a chemically synthesized derivative of rutin. It is produced by enzymatically attaching a glucose moiety to rutin to improve its water solubility and photostability. This chemical modification is inherent to AGR and is essential for its cosmetic and skincare applications; there are no additional chemical modifications beyond this initial synthesis [32]. Furthermore, the long-term safety of AGR remains insufficiently characterized, particularly with respect to chronic or high-dose exposure. AGR is classified as a potential skin sensitizer (Category 1), indicating that it may cause allergic skin reactions in predisposed individuals [33,34]. Additionally, AGR is regarded as toxic to aquatic life with long-lasting environmental impact (Chronic Aquatic Toxicity, Category 2), necessitating careful management of its release into the environment [33,34]. Notably, no other individual compounds—including flavonols, phenolic acids, or vitamin E—have demonstrated comparable standalone efficacy in PLE management [29]. This underscores a substantial unmet need for natural, safer, and environmentally sustainable alternatives to AGR that can achieve equivalent or superior photoprotective outcomes.

In pursuit of this goal, recent attention has turned to quercetin (QC) and hesperidin (HPN), two naturally derived flavonoids with potent antioxidant and anti-inflammatory properties [35,36,37]. QC, a yellow crystalline aglycone, is well recognized for its free radical scavenging ability and its protective effects against oxidative stress-induced cellular damage [35,38,39]. Its strong antioxidant activity is attributed to its polyhydroxylated structure and its conjugated double bond system [35,38,39]. QC has also been extensively studied for its anticancer, antiallergic, antidiabetic, anti-obesity, and antigout properties [37,39,40]. HPN, predominantly found in citrus fruits, has demonstrated protective effects against UV-induced skin damage by reducing inflammation, enhancing antioxidant defense mechanisms, and preventing keratinocyte apoptosis [36,41,42]. Preclinical models suggest that HPN effectively suppresses UVB-induced pro-inflammatory cytokine production and mitigates oxidative stress in skin cells [36,41,42]. In vitro studies indicate that QC and HPN exert synergistic antioxidant effects, protecting keratinocytes from UVB-induced apoptosis and ROS generation more effectively in combination than when applied individually [36,37,42]. Although the individual antioxidant properties of QC and HPN are well established, no previous studies have directly compared the photoprotective efficacy of the QC:HPN combination with AGR in photodermatoses models [43]. In particular, the synergistic photoprotective, anti-allergic, and anti-inflammatory effects of the optimized 8:1 QC:HPN combination have not been previously demonstrated or compared to clinically used agents such as AGR in PLE settings.

This study is the first to conduct a direct, head-to-head comparative assessment of QC:HPN combination versus AGR across multiple key parameters relevant to PLE pathogenesis, including cell viability, ROS production, pro-inflammatory cytokine expression, and DNA damage in an in vitro model of sun-induced skin allergy. Furthermore, through systematic formulation optimization, we identified and validated an 8:1 (*w*/*w*) QC:HPN ratio that demonstrates superior antioxidant efficacy, enhanced biocompatibility, and improved photoprotective capacity compared to AGR. To our knowledge, this is the first report to establish both the comparative advantage and the optimized ratio of this natural flavonoid combination, positioning it as a promising candidate for the development of safer and more effective photoprotective therapies.

The novelty of this study lies not only in the direct comparison with AGR but also in the identification of a natural, synergistic combination that may overcome the current limitations of synthetic photoprotective agents. Our findings provide critical insights into the potential of multi-targeted, flavonoid-based strategies to modulate oxidative stress and immune dysregulation in sun-induced skin allergies and related photodermatoses.

Ultimately, this work seeks to advance the field by contributing to the development of next-generation, natural compound-based photoprotection solutions that are not only effective and safe but also environmentally sustainable. By addressing the current therapeutic gaps, this study offers a meaningful step toward improving the management of PLE and enhancing the quality of life for affected individuals.

## 2. Materials and Methods

### 2.1. Cell Culture

Human dermal fibroblasts (PCS-201-010; ATCC, Manassas, VA, USA) and rat basophilic leukemia cells (RBL-2H3; CRL-2256; ATCC, Manassas, VA, USA) were cultured in Dulbecco’s Modified Eagle Medium (DMEM; Hyclone, SH30243.01; Logan, UT, USA) supplemented with 10% fetal bovine serum (FBS; Gibco, Grand Island, NY, USA) and 1% penicillin–streptomycin (Gibco, Grand Island, NY, USA) under standard culture conditions (37 °C, 5% CO_2_).

For the induction of photoallergic conditions and evaluation of photoprotective efficacy, human dermal fibroblasts were pre-incubated with QC (≥98% purity; Sigma-Aldrich, St. Louis, MO, USA), HPN (≥95% purity; Sigma-Aldrich, St. Louis, MO, USA), or AGR (≥95% purity; FUJIFILM Wako, Osaka, Japan) for 1 h.

Without removal of the compounds, the cells were then irradiated with HEV light at 41 J/cm^2^ in phenol red-free DMEM (Gibco, Grand Island, NY, USA), thereby allowing concurrent exposure to the test substances and HEV. RBL-2H3 cells were incubated in serum-free DMEM (Gibco, Grand Island, NY, USA) containing 100 ng/mL DNP-IgG (Sigma-Aldrich, St. Louis, MO, USA) for 24 h.

### 2.2. Preparation of Test Products

**Figure 1:**
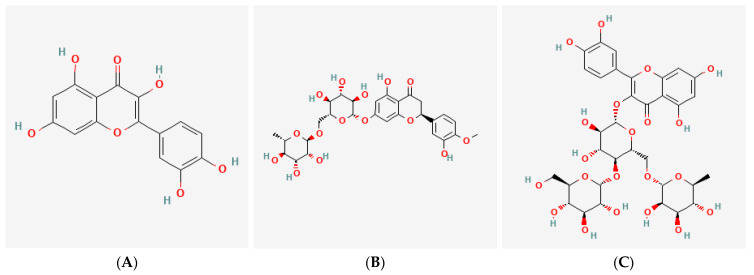
Chemical structures of the tested compounds: (**A**) Quercetin (C_15_H_10_O_7_), (**B**) Hesperidin (C_28_H_34_O_15_), (**C**) Alpha(α)-Glucosylrutin (C_27_H_32_O_16_, Quercetin with glucose attachment). The chemical structures were generated from PubChem database references.

QC, HPN, and AGR were used as test substances. The chemical structures of the three tested compounds can be found in Figure 1. Doses ranging from 2.5 to 160 μg/mL were selected based on prior reports of their photoprotective activity in dermal models, as well as preliminary cytotoxicity testing to determine the safe upper limits for each compound in human fibroblasts and RBL-2H3 mast cells. These concentrations allowed assessment of both efficacy and safety across a broad dynamic range. Test substances were first prepared at 100 times the highest treatment concentration and then diluted with culture medium before use. Depending on each compound’s solubility, either dimethyl sulfoxide (DMSO; Sigma-Aldrich, St. Louis, MO, USA) or sterile distilled water (Daihan Scientific, Wonju, Republic of Korea) was used as the solvent. The test formula containing QC:HPN 8:1 (weight ratio, *w*/*w*) used for ex vivo studies is referred to as ID Solar Defense Antevis Shield sunscreen (UCL Co., Ltd., Incheon, Republic of Korea), which is the finished product ready for consumer use. This formulation, containing the QC:HPN 8:1 (weight ratio, *w*/*w*) combination as the active component, is fully developed and is intended for commercial launch in multiple countries, including Korea as a cosmetic product and the United States as an over-the-counter product, in accordance with the regulatory requirements of each respective region. Separately, ID Aller-free™ is a raw material product that contains the same QC:HPN 8:1 combination, designed for use as an active ingredient in various cosmetic formulations. AGR (FUJIFILM Wako, Osaka, Japan) served as a positive control in this study. The reference concentration of 5 μg/mL was selected based on preliminary cytotoxicity assays, which indicated this dose was non-cytotoxic across all test groups and is commonly used in previous studies evaluating antioxidant and anti-photoaging effects of similar flavonoids [44].

### 2.3. Human Skin Tissue Preparation and Photoallergy Induction

Human skin tissue was obtained with the approval of the Institutional Review Board (IRB No. GIRB-24O29-HA; Global Medical Research Center, Seoul, Republic of Korea). All tissues were fully anonymized in accordance with IRB guidelines and the Declaration of Helsinki, ensuring no personally identifiable information was accessible to the researchers. The skin tissues were washed several times with phosphate-buffered saline (PBS; Gibco, Grand Island, NY, USA) to remove residual impurities and then cut into 1 cm × 1 cm sections for each test group. After application of the test product, tissues were cultured in a specialized medium (Gibco, Grand Island, NY, USA) at 37 °C under 5% CO_2_ conditions.

To induce photoallergic responses, the tissues were irradiated daily with either UVA at 12 J/cm^2^/day using a UVA lamp (Vilber Lourmat, Collégien, France) or HEV light at 68 J/cm^2^/day using a LED assay driver (LAD-1; Teleopto, Tokyo, Japan) and an irradiation system (Vilber Lourmat, Collégien, France).

### 2.4. Cell Culture Supernatant Collection and Protein Extraction

The culture medium from the treated cells was collected and centrifuged at 2000× *g* for 10 min. The supernatant, excluding cell debris, was used for subsequent analysis. Protein quantification was performed using a Bicinchoninic Acid (BCA) Protein Assay Kit (Sigma-Aldrich, St. Louis, MO, USA) according to the manufacturer’s instructions.

### 2.5. Cell Viability Assay

This assay was conducted to determine the appropriate, non-toxic concentrations of the test products when applied to cells. Cells were seeded at a density of 5 × 10^4^ cells per well in a 96-well plate (SPL Life Sciences, Pocheon, Republic of Korea). After reaching over 80% confluency, the test products were applied at various concentrations and incubated for 24 h.

Following treatment, the culture medium was replaced with WST substrate solution (CCK-8; Dojindo Laboratories, Kumamoto, Japan), and the cells were further incubated at 37 °C for 2 h. Absorbance was measured at 450 nm using a microplate reader (Varioskan LUX; Thermo Fisher Scientific, Waltham, MA, USA). Cell viability was calculated using the following formula:

$$\mathrm{Cell}\;\mathrm{viability}\;(\%)=\frac{ODexp-ODblnk}{ODcon-ODblnk}\times 100$$

- Blank (blnk): Medium containing only the WST substrate (blank).
- Control (con, (negative) control): Untreated control group.
- Experiment (exp): Experimental group treated with the test product.

### 2.6. Antioxidant Enzyme Activity Assay

This assay was conducted to evaluate the activity of the antioxidant enzymes superoxide dismutase (SOD) and catalase (CAT) in response to treatment with the test product. SOD and CAT activities were measured using the OxiTec™ SOD Assay Kit (BO-SOD-500; BIOMAX, Seoul, Republic of Korea) and the OxiTec™ Catalase Assay Kit (BO-CAT-400; BIOMAX, Seoul, Republic of Korea), respectively.

Human dermal fibroblasts were seeded at a density of 5 × 10^4^ cells per well in a 6-well plate (SPL Life Sciences, Pocheon, Republic of Korea) and cultured until they reached over 80% confluency. The test product was then applied at various concentrations, and the cells were incubated for 24 h.

The assay was performed in accordance with the manufacturer’s protocol. Absorbance was measured using a microplate reader (Varioskan LUX; Thermo Fisher Scientific, Waltham, MA, USA). The optical density (OD) values were analyzed according to the provided result analysis method. A lower absorbance value indicates higher antioxidant enzyme activity.

### 2.7. Assay for Anti-Allergic Activity

This assay was performed to evaluate the anti-allergic activity of the test product by measuring the secretion of β-hexosaminidase (β-hex), a biomarker released during allergic reactions. White rat basophilic leukemia cells (RBL-2H3; CRL-2256; ATCC, Manassas, VA, USA) were seeded at a density of 2 × 10^5^ cells per well in a 24-well plate (SPL Life Sciences, Pocheon, Republic of Korea) and subjected to allergic stimulation.

After allergy induction, the cells were washed twice with PIPES buffer (Sigma-Aldrich, St. Louis, MO, USA) and treated with various concentrations of the test product in PIPES buffer containing 4 mM MgCl_2_ (Sigma-Aldrich, St. Louis, MO, USA), 5.6 mM glucose (Sigma-Aldrich, St. Louis, MO, USA), and 0.1% bovine serum albumin (BSA; Sigma-Aldrich, St. Louis, MO, USA). The cells were incubated at 37 °C in a 5% CO_2_ incubator for 1 h, followed by stimulation with 100 ng/mL DNP-BSA (Sigma-Aldrich, St. Louis, MO, USA) for an additional 1 h. The reaction was terminated by placing the cells on ice for 10 min.

Subsequently, 20 μL of the cell supernatant was transferred to a 96-well plate (SPL Life Sciences, Pocheon, Republic of Korea), followed by the addition of 80 μL of 1 mM p-nitrophenyl-N-acetyl-β-D-glucosaminide (Sigma-Aldrich, St. Louis, MO, USA). After incubation at 37 °C for 1 h, 200 μL of stop solution (0.1 M NaHCO_3_ and 0.1 M Na_2_CO_3_; Sigma-Aldrich, St. Louis, MO, USA) was added to each well to terminate the reaction. Absorbance was measured at 405 nm using a microplate reader (Varioskan LUX; Thermo Fisher Scientific, Waltham, MA, USA). β-hexosaminidase secretion was calculated using the following equation:

$$\beta -\mathrm{hex}\;\mathrm{secretion}\;(\%)=\frac{ODexp}{ODcon}\times 100$$

- Control (con): Cells treated without the test product (negative control).
- Experiment (exp): Cells treated with DNP-IgG and the test product.

### 2.8. Cytokine Production Assay

This assay was conducted to evaluate cytokine production following treatment with the test product in cells and human skin tissues. White rat basophilic leukemia cells (RBL-2H3; CRL-2256; ATCC, Manassas, VA, USA) were seeded at a density of 5 × 10^4^ cells per well in a 6-well plate (SPL Life Sciences, Pocheon, Republic of Korea) and incubated with various concentrations of the test product for 1 h. Allergic stimulation was induced by treatment with 100 ng/mL DNP-BSA (Sigma-Aldrich, St. Louis, MO, USA) for 4 h. Subsequently, the cells were placed on ice for 10 min to terminate the reaction.

Cytokine levels in the culture supernatant of RBL-2H3 cells were quantified using a Rat IL-6 ELISA Kit (R6000B; R&D Systems, Minneapolis, MN, USA) and a Rat TNF-α ELISA Kit (KRC3001; Invitrogen, Carlsbad, CA, USA). For human skin tissue samples, cytokine expression was analyzed from protein extracts using the following kits: Human IL-1β ELISA Kit High Sensitivity (A323168; Antibodies, Davis, CA, USA), Human IL-2 High Sensitivity ELISA Kit (BMS221-SHS; Invitrogen, Carlsbad, CA, USA), Human IL-6 ELISA Kit High Sensitivity (AB46042; Abcam, Cambridge, UK), and Human TNF-α ELISA Kit (KHC3014; Invitrogen, Carlsbad, CA, USA).

All procedures were performed according to the manufacturers’ protocols. Absorbance was measured using a microplate reader (Varioskan LUX; Thermo Fisher Scientific, Waltham, MA, USA). Cytokine concentrations were calculated by substituting the optical density (OD) values into a regression equation derived from the standard curve. An increase in absorbance indicated a higher concentration of the corresponding cytokine.

### 2.9. Histological Staining

For histological evaluation, tissue samples were collected 72 h after application of the test product and fixed in 10% formalin. Paraffin-embedded blocks were prepared, and sections were cut and mounted on slides. Paraffin was removed, and the tissue sections were stained following the protocol described. After staining, the tissues were thoroughly dehydrated through a water washing process and then mounted using mounting solution.

The epidermis and dermis of the fixed tissues were observed under an optical microscope (DM3000LED; Leica Microsystems, Wetzlar, Germany). Collagen fibers were stained blue and elastin fibers black using the Verhoeff–Van Gieson staining method, following the protocol [45].

The total area of the dermis, collagen fiber area, and elastin fiber area were quantified from the tissue images using an image analysis program (ImageJ; National Institutes of Health, Bethesda, MD, USA).

### 2.10. Masson’s Trichrome (MT) Staining

This assay was performed to evaluate collagen fiber production in human skin tissues. Tissue samples were fixed and processed with Bouin’s solution (2010; BBC Biochemical, Mount Vernon, WA, USA) and then washed under running water. Sections were stained with Weigert’s iron hematoxylin solution, prepared using hematoxylin (4077-4425; Daejung, Siheung, Republic of Korea) and ferric chloride (660; Duksan, Ansan, Republic of Korea), followed by another washing step.

The cytoplasm and muscle fibers were stained with Biebrich scarlet acid fuchsin solution, prepared using Biebrich scarlet (B6008; Sigma-Aldrich, St. Louis, MO, USA) and acid fuchsin (4048-4125; Daejung, Siheung, Republic of Korea). Sections were then mordanted and decolorized using phosphomolybdic-phosphotungstic acid solution, prepared using phosphomolybdic acid hydrate (84235S0410; Junsei, Tokyo, Japan) and phosphotungstic acid hydrate (84220S0410; Junsei, Tokyo, Japan).

Subsequently, collagen fibers were stained with aniline blue (1087-4125; Daejung, Siheung, Republic of Korea) without washing, and unbound aniline blue was removed by treatment with acetic acid (Sigma-Aldrich, St. Louis, MO, USA) for decolorization. The collagen fiber production was quantified as the percentage of collagen fiber area relative to the total dermal area using the following equation:

$$\mathrm{Collagen}\;\mathrm{fiber}\;\mathrm{production}\;(\%)=\frac{Collagen\;fiber\;area}{Total\;area\;of\;dermis}\times 100$$

Higher collagen fiber production indicates a greater presence of collagen fibers within the tissue cross-section.

### 2.11. Verhoeff–Van Gieson (VVG) Staining

This assay was performed to evaluate elastin fiber production in human skin tissues. Tissue sections were stained with Verhoeff’s solution (HT25A; Sigma-Aldrich, St. Louis, MO, USA), followed by bleaching with 2% ferric chloride (451649; Sigma-Aldrich, St. Louis, MO, USA), which was freshly prepared before use. After bleaching, tissues were washed and iodine was removed using a 5% sodium thiosulfate solution (S8503; Sigma-Aldrich, St. Louis, MO, USA), also freshly prepared prior to use. Subsequently, the sections were counterstained with Van Gieson solution (HT25B; Sigma-Aldrich, St. Louis, MO, USA) after a further washing step. Elastin fiber production was calculated as the percentage of elastin fiber area relative to the total dermal area using the following formula:

$$\mathrm{Elastin}\;\mathrm{fiber}\;\mathrm{production}\;(\%)=\frac{Elastin\;fiber\;area}{Total\;area\;of\;dermis}\times 100$$

A higher elastin fiber production indicates a greater presence of elastin fibers within the tissue cross-section.

### 2.12. Statistical Analysis

All assays were performed using a minimum of three independent biological replicates (n = 3), and for each biological replicate, three technical repeats were conducted to ensure the reliability and statistical validity of the results. Results are expressed as the mean ± standard error of the mean (SEM). Statistical analyses were performed using IBM SPSS Statistics (version 25.0; IBM Corp., Armonk, NY, USA). For normally distributed data, comparisons between two groups were performed using a paired samples *t*-test. For multiple group comparisons, one-way ANOVA followed by Tukey’s HSD post hoc test was used. A *p*-value < 0.05 was considered statistically significant.

## 3. Results

### 3.1. Evaluation of Cell Viability

**Figure 2:**
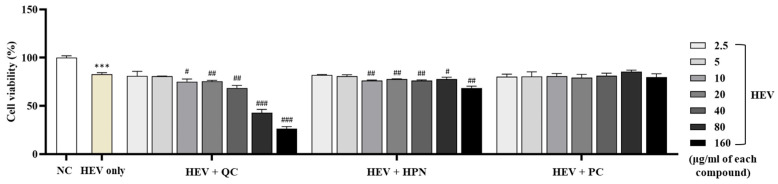
Cell viability of human dermal fibroblasts following exposure to high-energy visible (HEV) light (41 J/cm^2^). Quercetin (QC), Hesperidin (HPN), and α-Glucosylrutin (AGR, positive control) were applied at various concentrations (2.5–160 µg/mL) to assess their protective effects. Treatment with these compounds significantly improved cell viability compared to the HEV-only group (# *p* < 0.05, ## *p* < 0.01, ### *p* < 0.001). AGR exhibited the most pronounced protective effect, restoring viability close to or above the untreated control level. *** *p* < 0.001 vs. NC.

Exposure to high-energy visible (HEV) light (400–500 nm) induces oxidative stress and cytotoxicity in human dermal fibroblasts, consistent with studies showing HEV-generated ROS reduces cell viability and extracellular matrix integrity [46]. Figure 2 summarizes the cell viability of human dermal fibroblasts exposed to HEV.

Exposure of human dermal fibroblasts to HEV light significantly decreased cell viability compared to the untreated negative control group (NC), confirming HEV-induced cytotoxicity. HEV exposure decreased fibroblast viability to 82.74 ± 1.696% compared to the negative control group (100.00 ± 1.889%, *p* < 0.001). This confirms the cytotoxic effect of HEV in the present experimental setting.

To evaluate the protective effects of antioxidant compounds, fibroblasts were treated with QC, HPN, and AGR at concentrations ranging from 2.5 to 160 μg/mL concurrently with HEV exposure.

QC exhibited a concentration-dependent dual behavior. At low to moderate concentrations (2.5–20 μg/mL), QC modestly preserved cell viability (80.92 ± 4.858%, 80.76 ± 0.214%, 75.02 ± 2.803%, and 75.46 ± 0.922%, respectively), showing no statistical significance compared to the HEV-only group. However, at higher concentrations (≥40 μg/mL), QC markedly decreased viability (68.55 ± 2.648%, 42.99 ± 3.285%, and 26.48 ± 2.131% at 40, 80, and 160 μg/mL, respectively), with significance levels of ## *p* < 0.01 and ### *p* < 0.001 versus HEV-only, indicating dose-dependent cytotoxicity, reflecting dose-dependent toxicity observed in other flavonoids like apigenin [47].

HPN treatment demonstrated consistent biocompatibility across all tested concentrations, with fibroblast viability ranging from 82.06 ± 0.546% at 2.5 μg/mL to 68.41 ± 2.044% at 160 μg/mL. Notably, there were no statistically significant reductions in viability relative to the HEV-only group, indicating a stable protective effect even at higher doses. This trend aligns with previous findings that HPN exerts antioxidative effects by reducing ROS and lipid peroxidation, particularly in neuronal cells [48,49]. Furthermore, HPN showed excellent safety in immune-related cell lines, maintaining RBL-2H3 viability above 94% at all concentrations tested [49]. These results support the broad-range applicability of HPN as a non-cytotoxic antioxidant suitable for dermal and systemic use.

AGR exhibited the strongest protective effects among the tested compounds. Fibroblast viability was consistently maintained or enhanced across all concentrations, reaching a peak of 85.42 ± 1.687% at 80 μg/mL (*p* < 0.01 vs. HEV-only), which notably exceeded the viability of the untreated control group. Even at the highest tested concentration of 160 μg/mL, AGR sustained high cell viability above 79%, indicating minimal cytotoxicity and excellent dose tolerance.

Collectively, these results highlight that while AGR exhibits the most robust and dose-tolerant photoprotective activity, QC requires cautious dose optimization due to its narrow therapeutic range. Based on these findings, QC concentrations at or below 20 μg/mL were identified as appropriate for combination use with HPN to avoid cytotoxicity and retain protective efficacy under HEV exposure.

**Figure 3:**
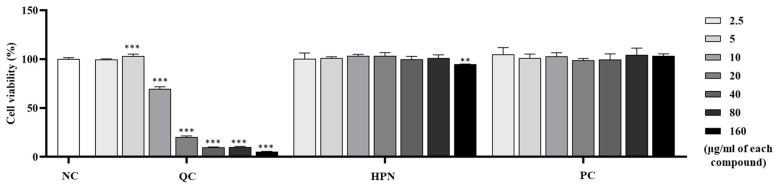
Cell viability of RBL-2H3 cells following treatment with Quercetin (QC), Hesperidin (HPN), and α-Glucosylrutin (AGR, positive control) at concentrations ranging from 2.5 to 160 µg/mL. QC treatment resulted in dose-dependent cytotoxicity, whereas HPN and AGR maintained high cell viability across all tested concentrations. Statistical significance is indicated relative to the negative control group (** *p* < 0.01, *** *p* < 0.001).

To evaluate the cytotoxicity of test compounds on immune-related cells, the viability of RBL-2H3 white rat basophilic leukemia cells was assessed after treatment with QC, HPN, and AGR at concentrations ranging from 2.5 to 160 μg/mL. Figure 3 illustrates the cell viability of RBL-2H3 cells treated with QC, HPN, and AGR.

QC treatment showed a clear dose-dependent cytotoxicity in RBL-2H3 cells. While low concentrations (2.5 and 5 μg/mL) maintained cell viability comparable to control (99.62 ± 0.677% and 103.19 ± 1.988%, respectively), higher concentrations resulted in a drastic reduction: 69.57 ± 2.155% at 10 μg/mL (*p* < 0.001), 20.30 ± 1.163% at 20 μg/mL (*p* < 0.001), 9.80 ± 0.445% at 40 μg/mL, 10.15 ± 0.605% at 80 μg/mL, 5.45 ± 0.291% at 160 μg/mL, all *p* < 0.001 vs. NC. This sharp decline indicates strong dose-dependent cytotoxicity of QC in immune cell types, especially at concentrations above 10 μg/mL. Induced severe cytotoxicity in RBL-2H3 basophils at ≥10 µg/mL (viability ≤ 69.57%), emphasizing caution in formulations targeting immune-related cells [50].

HPN demonstrated excellent biocompatibility across the entire concentration range in RBL-2H3 basophilic leukemia cells. Cell viability remained consistently high, exceeding 94% at all tested doses, from 100.28 ± 5.992% at 2.5 μg/mL to 94.81 ± 0.239% at 160 μg/mL. No statistically significant cytotoxic effects were observed, showing that HPN is well-tolerated and potentially suitable for both systemic and dermal applications. Similarly, AGR exhibited high viability and biocompatibility in immune cells, with viability values exceeding 100% across most concentrations—104.91 ± 6.866% at 2.5 μg/mL, 101.10 ± 4.119% at 5 μg/mL, 102.94 ± 3.637% at 10 μg/mL, and 103.48 ± 1.945% at 160 μg/mL. No cytotoxic responses were detected at any concentration tested, reaffirming AGR’s favorable safety profile, which aligns with existing clinical data supporting its use in dermatological applications [32].

Overall, these findings indicate that although QC exhibits significant protective effects against HEV-induced oxidative damage in dermal fibroblasts, its application is limited by potential immunotoxicity at elevated concentrations. Therefore, careful dose optimization is essential. The data support the use of QC at concentrations ≤ 5 μg/mL, particularly in combination with HPN, which demonstrated stable biocompatibility and favorable performance across both dermal and immune cell models.

Mechanistic Insights and Therapeutic Implications: HEV-induced oxidative stress is associated with ROS generation at levels comparable to approximately 25% of UVA-induced oxidative capacity [46]. This highlights the necessity for effective antioxidants—such as AGR—to counteract HEV-related photoaging and cellular damage [32]. Similarly to celastrol’s dose-dependent immunotoxicity [50], QC requires precise concentration control (≤5 µg/mL) to balance efficacy and safety. HPN’s stability and AGR’s potency show synergistic utility in topical formulations, while QC’s therapeutic window limits its use to low-dose adjunctive roles. These results underscore the importance of compound-specific profiling for dermatological and immunomodulatory applications, particularly in mitigating HEV-associated photodamage.

### 3.2. Antioxidant Efficacy Evaluation

To investigate the antioxidative potential of QC and HPN combinations under HEV light-induced oxidative stress, the activities of two key antioxidant enzymes—SOD and CAT—were measured. Exposure to HEV (blue light) induces oxidative stress by generating ROS, which are detoxified by endogenous enzymes such as SOD and CAT [51]. SOD converts superoxide radicals to hydrogen peroxide, which is then broken down by CAT to water and oxygen [51]. Deficits in these enzymes lead to cellular damage, as seen in the HEV-exposed group.

**Figure 4:**
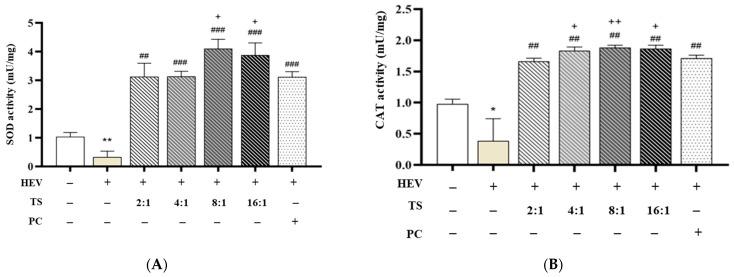
Antioxidant enzyme activity in fibroblasts following high-energy visible (HEV) light exposure (41 J/cm^2^). (**A**) Superoxide dismutase (SOD) activity and (**B**) catalase (CAT) activity were measured after treatment with quercetin/hesperidin (QC:HPN) mixtures at various ratios (2:1, 4:1, 8:1, and 16:1). The total flavonoid concentration was consistently maintained at 5 µg/mL across all test groups. α-Glucosylrutin (AGR, 5 µg/mL) was used as the positive control (PC) for direct comparison. Statistical significance: * *p* < 0.05, ** *p* < 0.01 vs. negative control (NC); ## *p* < 0.01, ### *p* < 0.001 vs. HEV-only group; + *p* < 0.05, ++ *p* < 0.01 vs. PC group (independent samples *t*-test).

Figure 4 shows the antioxidant enzyme activity in fibroblasts following exposure to HEV. Figure 4A represents SOD activity measurement, and Figure 4B represents CAT activity measurement. Human dermal fibroblasts exposed to HEV light exhibited significantly reduced enzymatic activity (SOD: 0.337 ± 0.199, CAT: 0.387 ± 0.355 mU/mg), consistent with previously reported oxidative damage profiles associated with blue light irradiation [52].

Treatment with AGR (5 μg/mL), used as a positive control, effectively restored enzyme activity (SOD: 3.127 ± 0.181, CAT: 1.714 ± 0.048, ### *p* < 0.001), corroborating its known efficacy as a ROS scavenger [53].

QC is recognized as one of the most potent natural flavonoids for upregulating the endogenous antioxidant defense system. It activates the Nrf2/Keap1 pathway, promoting the transcription of antioxidant response elements and enhancing the activities of SOD and CAT [54,55]. This mechanism is consistent with the observed restoration and enhancement of enzyme activities in QC:HPN-treated fibroblasts. HPN, while less potent as a direct ROS scavenger, is known for its anti-inflammatory and vascular protective roles, and it also contributes to antioxidant defense by modulating cellular redox status and supporting enzyme activities, albeit to a lesser extent than QC [56,57]. Remarkably, various QC:HPN ratio combinations—while maintaining a constant total concentration of 5 μg/mL—demonstrated differential enhancements in enzyme activity, showing that the antioxidative efficacy of the formulation is strongly dependent on the proportion of QC to HPN.

Among the tested ratios, the 8:1 (QC:HPN) treatment showed the most pronounced effect, with SOD activity reaching 4.112 ± 0.320 and CAT 1.885 ± 0.037, significantly exceeding both the HEV-only group (*p* < 0.001) and the AGR group. The 16:1 ratio also demonstrated comparably high activity (SOD: 3.886 ± 0.420, CAT: 1.868 ± 0.034), while lower QC ratios (2:1, 4:1) elicited intermediate effects (e.g., 2:1 → SOD: 3.136 ± 0.461, CAT: 1.665 ± 0.048). The optimal QC:HPN ratio (8:1) identified in the experiment is consistent with the principle that QC’s stronger antioxidant activation is maximized when it is the predominant component, while HPN supports biocompatibility and anti-inflammatory effects [58,59]. This ratio balances efficacy and safety, as both compounds are well-tolerated at the tested concentrations.

These findings align with the established mechanisms of QC, a flavonoid known to upregulate Nrf2-mediated antioxidant response elements and directly scavenge superoxide and hydrogen peroxide species [60,61]. HPN, although possessing mild antioxidant properties, is reported to function more effectively in anti-inflammatory and vascular protective roles rather than as a potent ROS quencher [62]. Therefore, increasing the relative concentration of QC within the combination formula appears to synergistically maximize intracellular enzymatic defense against HEV-induced oxidative stress.

Importantly, all tested QC:HPN ratios showed significant improvement over HEV-only conditions without compromising cell viability, supporting the biocompatibility of the tested combinations. Taken together, these data support the selection of a QC:HPN ratio of 8:1 as the optimal condition for downstream formulation development, balancing antioxidant efficacy, cellular compatibility, and formulation feasibility. This ratio is thus proposed as the most appropriate benchmark for future preclinical or clinical translation.

### 3.3. Evaluation of Anti-Allergic Efficacy

**Figure 5:**
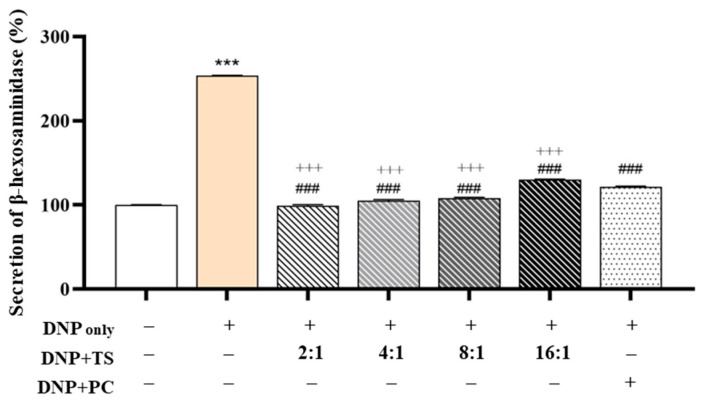
Inhibitory effects of QC:HPN combinations on β-hexosaminidase release in DNP-stimulated RBL-2H3 cells. Cells were treated with QC:HPN mixtures at various ratios (2:1, 4:1, 8:1, and 16:1), maintaining a constant total flavonoid concentration of 5 µg/mL. α-Glucosylrutin (AGR, 5 µg/mL) served as the positive control (PC). Bar colors represent different treatment groups. Statistical significance: *** *p* < 0.001 vs. negative control (NC); ### *p* < 0.001 vs. DNP-only group; +++ *p* < 0.001 vs. AGR group (independent samples *t*-test).

Optimized QC:HPN (8:1) Ratio Shows Superior Inhibition of β-Hexosaminidase Release Compared to AGR: To investigate the anti-allergic potential of QC and HPN (HPN) combinations, we evaluated their effects on β-hexosaminidase (β-hex) release in DNP-stimulated RBL-2H3 basophilic cells, a validated model for mast cell degranulation (Figure 5). Stimulation with DNP markedly elevated β-hex secretion from the baseline (100.000 ± 0.493%) to 253.795 ± 0.360% (*p* < 0.001), confirming robust activation of allergic pathways.

The positive control, AGR (5 μg/mL), partially reversed this increase, reducing β-hex release to 121.325 ± 1.145% (### *p* < 0.001 vs. DNP), consistent with prior findings on its mast cell-stabilizing properties.

Notably, among all tested QC:HPN ratios (total concentration fixed at 5 μg/mL), the 8:1 combination produced the strongest inhibitory effect, reducing β-hex release to 99.021 ± 1.450%—a level not only significantly lower than the DNP group but also more effective than AGR, which failed to fully normalize β-hex levels. The 4:1 combination also demonstrated strong efficacy (105.022 ± 1.399%), but the 8:1 ratio consistently showed the closest return to baseline, indicating an optimal synergistic balance.

This enhanced effect of the 8:1 ratio is likely driven by the dominant presence of QC, a flavonoid known to directly interfere with the FcεRI-mediated signaling cascade in mast cells. QC has been shown to inhibit key kinases such as Lyn and Syk, thereby suppressing calcium influx and granule exocytosis. This is consistent with studies demonstrating QC’s direct inhibition of FcεRI signaling through the suppression of Lyn and Syk, which are essential for these cellular processes [63,64]. QC binds CLM-1 (*K*~D~ = 2.962 × 10^−5^ mol/L), triggering SHP-1 phosphorylation and downstream suppression of MyD88/NF-κB signaling [63]. This pathway mirrors its inhibition of FcεRI-mediated degranulation in the current study. QC reduces MRGPRX2 membrane expression by inhibiting F-actin polymerization via PI3K/AKT/Rac1/Cdc42 [63], a mechanism consistent with its normalization of β-hex levels in the 8:1 formulation.

In contrast, HPN appears to have complementary antioxidant and anti-inflammatory properties, though its direct effect on mast cell degranulation is relatively limited. By enhancing cellular resilience through its antioxidant activity, HPN complements QC’s targeted inhibition of kinases, resulting in a synergistic effect that is not achieved by either compound alone [65].

Notably, these findings demonstrate that an 8:1 ratio of QC to HPN (QC:HPN) at a fixed concentration of 5 μg/mL is more effective than AGR, offering valuable guidance for formulation optimization. This specific ratio restores β-hexosaminidase secretion to baseline levels seen in non-activated cells, without requiring an increased total compound dose. In contrast, while AGR partially suppresses β-hex release (121.33%), its role as a ROS scavenger lacks the direct kinase inhibition exhibited by QC, which likely accounts for its reduced efficacy despite its prior use as a positive control.

### 3.4. Inhibitory Effects of the Test Product on Pro-Inflammatory Cytokine Production (IL-6 and TNF-α)

**Figure 6:**
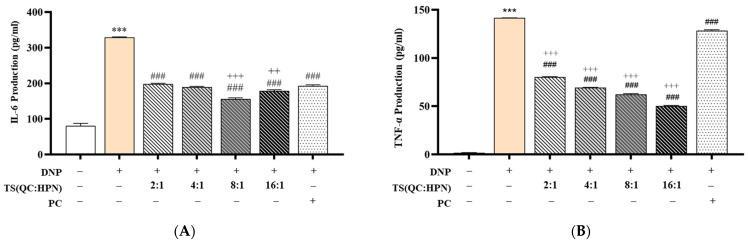
Effects of QC:HPN treatment on cytokine secretion in DNP-stimulated RBL-2H3 cells. (**A**) Interleukin-6 (IL-6) production and (**B**) tumor necrosis factor-alpha (TNF-α) production were measured after treatment with quercetin/hesperidin (QC:HPN) mixtures at various ratios (2:1, 4:1, 8:1, and 16:1). The total flavonoid concentration was consistently maintained at 5 µg/mL across all test groups. α-Glucosylrutin (AGR, 5 µg/mL) was used as the positive control (PC) for direct comparison. Bar colors represent different treatment groups. Statistical significance: *** *p* < 0.001 vs. negative control (NC); ### *p* < 0.001 vs. DNP-only group; ++ *p* < 0.01, +++ *p* < 0.001 vs. PC group (independent samples *t*-test).

To evaluate the anti-inflammatory efficacy of the QC:HPN complex, cytokine levels of IL-6 and TNF-α were measured in DNP-stimulated RBL-2H3 cells (Figure 6). DNP treatment significantly increased IL-6 (328.91 ± 1.48 pg/mL) and TNF-α (141.58 ± 0.15 pg/mL) production compared to the normal control (NC) group (IL-6: 80.78 ± 6.87 pg/mL; TNF-α: 1.87 ± 0.06 pg/mL) (*p* < 0.001, vs. NC).

QC:HPN treatment significantly suppressed both IL-6 and TNF-α levels in a dose-dependent manner across all tested ratios (2:1, 4:1, 8:1, 16:1). Among these, the 8:1 QC:HPN formulation exhibited the most pronounced inhibition, reducing IL-6 to 155.95 ± 3.16 pg/mL and TNF-α to 62.33 ± 0.72 pg/mL. This suppression was statistically significant when compared to the DNP-only group (*p* < 0.001) as well as the positive control AGR (*p* < 0.001 vs. AGR), which showed IL-6 and TNF-α levels of 193.28 ± 2.71 pg/mL and 128.47 ± 0.85 pg/mL, respectively. This aligns with studies showing QC’s ability to inhibit NF-κB nuclear translocation and suppress pro-inflammatory gene expression [55,66,67].

The anti-inflammatory effect of QC:HPN displayed a non-linear dose–response profile, with the 8:1 ratio showing superior activity compared to both lower (2:1, 4:1) and higher (16:1) ratios. This bell-shaped response shows a saturation threshold or feedback regulation commonly seen in complex phytocompounds or protein-based formulations.

Importantly, QC:HPN at 8:1 not only decreased cytokine levels more effectively than AGR but also approached near-baseline levels of IL-6 and TNF-α observed in the NC group. The bell-shaped efficacy curve (peak at 8:1 ratio) mirrors observations in flavonoid studies, where higher concentrations may saturate cellular targets or induce feedback inhibition [67,68]. For example, QC’s dual role as an antioxidant and pro-oxidant at varying doses influences its cytokine modulation [55]. These findings indicate that QC:HPN at this specific ratio may exert a dual action by both preventing hyperactivation of immune signaling and potentially restoring immune homeostasis.

Given the central role of IL-6 and TNF-α in type I hypersensitivity and allergic inflammation, the observed suppression supports the potential of QC:HPN as a strong candidate for managing allergic or inflammatory conditions. The results further imply that its mechanism may involve modulation of pro-inflammatory signaling pathways such as NF-κB or MAPK cascades, which warrants further mechanistic studies.

### 3.5. Effects of the Test Product on HEV-Induced Cytokine Production in Human Skin Tissue

HEV light, especially within the blue light spectrum (400–500 nm), is known to penetrate deeply into the skin and trigger oxidative stress by activating photoreceptors and promoting intracellular generation of ROS, thereby initiating inflammatory responses in skin cells [46,69,70]. This oxidative stress activates photoreceptors and intracellular signaling pathways, such as NF-κB, leading to the upregulation of key pro-inflammatory cytokines—including IL-1β, IL-6, and TNF-α—as observed in both keratinocytes and fibroblasts [70]. The results from the HEV Only group, which showed marked increases in IL-1β, IL-2, IL-6, and TNF-α, are consistent with prior studies indicating that HEV light exposure causes dermal inflammation and matrix degradation [69,70].

**Figure 7:**
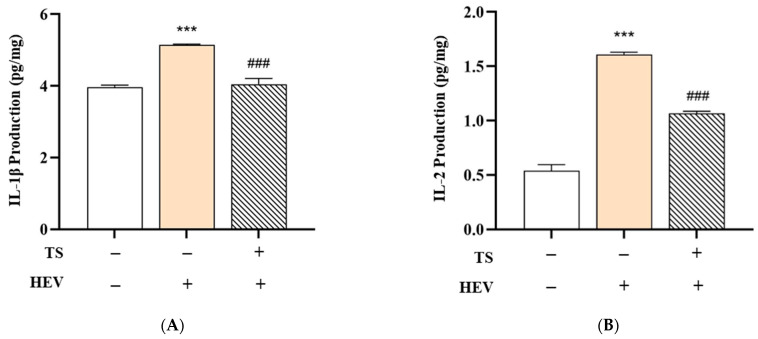

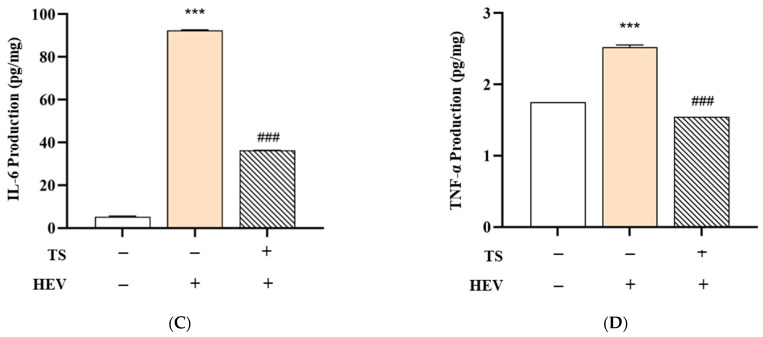
Effects of QC:HPN (8:1) on HEV-induced cytokine production in human skin tissue. Human skin tissues were exposed to high-energy visible (HEV) light (68 J/cm^2^) to induce inflammatory cytokine expression. Test samples (TS) containing QC:HPN (8:1) at a total concentration of 5 µg/mL were applied after exposure. (**A**) Interleukin-1β (IL-1β) production, (**B**) IL-2 production, (**C**) IL-6 production, and (**D**) tumor necrosis factor-α (TNF-α) production were measured. Bar colors represent different treatment groups. Statistical significance: *** p < 0.001 vs. negative control (untreated); ### *p* < 0.001 vs. HEV-only group (vehicle control) (independent samples *t*-test).

To evaluate the protective efficacy of QC:HES (8:1) included formular against HEV-induced skin inflammation, we measured the levels of key pro-inflammatory cytokines—IL-1β, IL-2, IL-6, and TNF-α—in human skin tissue subjected to three treatment conditions: Negative Control (no HEV exposure), HEV Only (HEV exposure without test product), and HEV + Test Product (HEV exposure with test product application). The data are summarized in Figure 7.

In the HEV Only group, a significant upregulation of all measured cytokines was observed compared to the Negative Control group. Specifically, levels of IL-1β increased from 3.97 ± 0.05 to 5.14 ± 0.03 pg/mg, IL-2 from 0.54 ± 0.06 to 1.61 ± 0.02 pg/mg, IL-6 from 5.36 ± 0.27 to 92.49 ± 0.22 pg/mg, and TNF-α from 1.75 ± 0.00 to 2.52 ± 0.03 pg/mg (*** *p* < 0.001 for all). These findings indicate that HEV radiation activates the NF-κB signaling pathway, resulting in increased expression of inflammatory cytokines and degradation of the dermal matrix.

Remarkably, treatment with QC:HES (8:1), including formulation, significantly mitigated this cytokine surge. In the HEV + Test Product group, the levels of IL-1β (4.05 ± 0.16 pg/mg), IL-2 (1.07 ± 0.02 pg/mg), IL-6 (36.31 ± 0.07 pg/mg), and TNF-α (1.54 ± 0.00 pg/mg) were all significantly reduced compared to the HEV Only group (### *p* < 0.001), indicating that the test product effectively suppressed the inflammatory response.

ROS Scavenging: Both QC and HPN are established antioxidants that neutralize free radicals generated by HEV light, thereby reducing oxidative stress and downstream inflammatory signaling [41,71,72]. NF-κB and MAPK Pathway Inhibition: QC has been shown to suppress NF-κB and MAPK signaling, which are central to the upregulation of pro-inflammatory cytokines such as IL-6 and TNF-α [41,72]. HPN also inhibits MAPK-dependent signaling, further contributing to the anti-inflammatory effect [41]. However, we acknowledge that the present study did not include direct mechanistic validation using protein or gene expression analyses (e.g., Western blot or qPCR). Further studies are required to verify the proposed involvement of these pathways.

Epidermal Barrier Stabilization: By reducing oxidative damage and inflammation, these flavonoids help maintain skin barrier integrity, which is crucial for preventing chronic inflammatory skin conditions [71,72]. This anti-inflammatory effect may be attributed to the inclusion of antioxidant and photoprotective agents in the formulation, which potentially act by scavenging HEV-induced ROS or by stabilizing epidermal barrier function. Previous reports have demonstrated that HEV-targeted sunscreens containing specific filters, such as iron oxides or botanical antioxidants (e.g., ferulic acid, niacinamide), significantly reduce HEV-induced hyperpigmentation and inflammatory marker expression in both in vitro and in vivo models.

Collectively, these findings support the notion that QC:HES (8:1) included formular confers robust protection against HEV-mediated skin damage by attenuating cytokine-mediated inflammation. This showed its potential utility in formulations designed for daily photoprotection in environments rich in digital blue light exposure.

### 3.6. Effects of PDRN and L-PDRN on no Production Under Non-Inflammatory and Inflammatory Conditions

**Figure 8:**
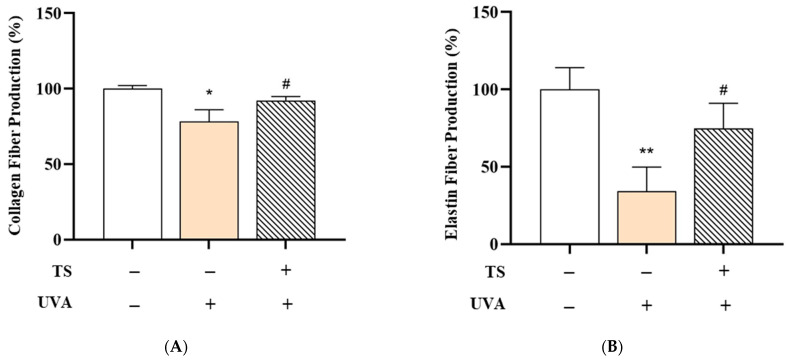
Effects of QC:HPN (8:1) on UVA-induced reduction in collagen and elastin fiber production in human skin tissue. Human skin tissues were exposed to UVA (dose not specified) with or without subsequent treatment with the test sample (TS) containing QC:HPN (8:1) at a total concentration of 5 µg/mL. (**A**) Collagen fiber production and (**B**) elastin fiber production were measured and expressed as percentages relative to the untreated control (NC, set to 100%). Statistical significance: * p < 0.05, ** p < 0.01 vs. negative control; # *p* < 0.05 vs. UVA-only (vehicle) group (independent samples *t*-test).

**Figure 9:**
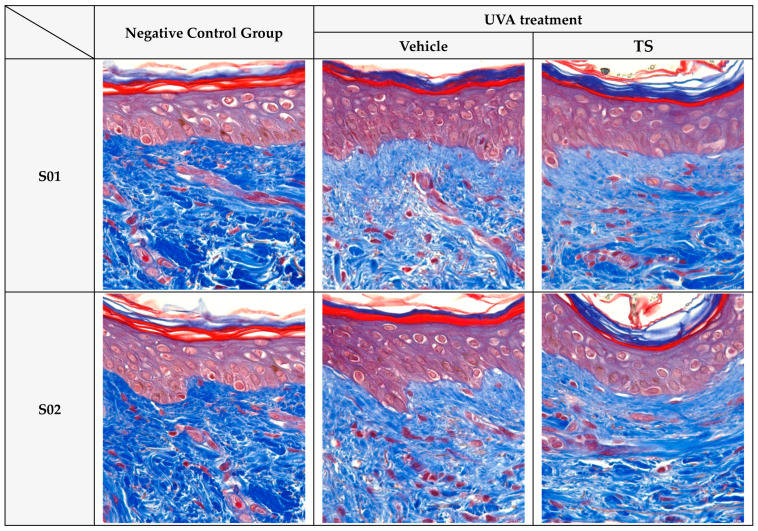

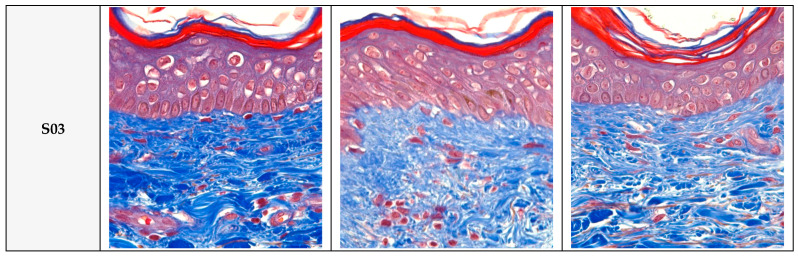
Protective effects of QC:HPN (8:1) on collagen structure in UVA-exposed human skin tissue (Masson’s trichrome staining, 400×). Representative images of human skin tissue cross-sections stained with Masson’s trichrome (MT) at 400× magnification. Tissues were divided into three groups: untreated negative control, UVA-irradiated vehicle control, and QC:HPN (8:1)-treated test sample (TS) group. In the vehicle group, UVA exposure resulted in significant collagen degradation and disruption of dermal architecture. In contrast, treatment with QC:HPN (8:1) preserved collagen fiber density and improved tissue structure. Collagen fibers are stained blue, keratin and cytoplasm are stained red, and nuclei are stained dark. S01–S03 represent tissue sections from three independent human donors.

**Figure 10:**
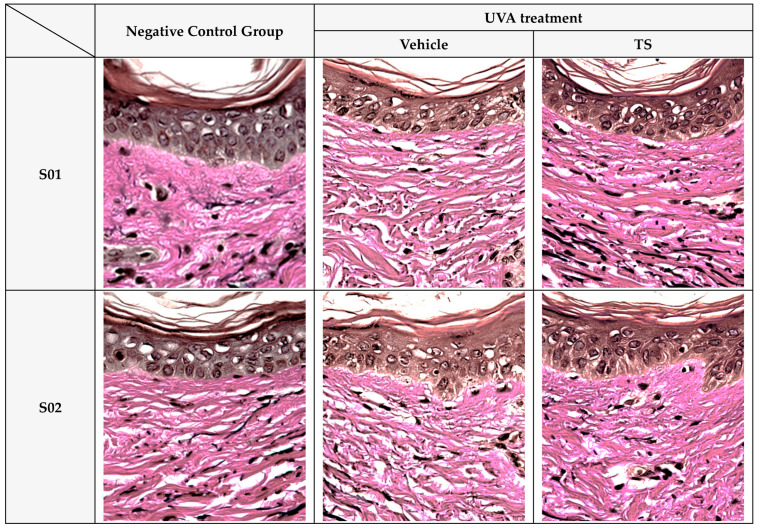

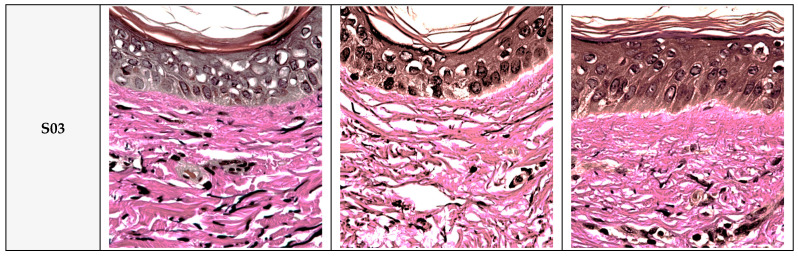
Protective effects of QC:HPN (8:1) on elastin fiber structure in UVA-exposed human skin tissue (Verhoeff–Van Gieson staining, 400×). Representative images of human skin tissue cross-sections stained with Verhoeff–Van Gieson (VVG) at 400× magnification. Tissues were categorized into three groups: untreated negative control, UVA-irradiated vehicle control, and QC:HPN (8:1)-treated test sample (TS) group (5 µg/mL). VVG staining visualizes elastin fibers in black, collagen in red, and cytoplasm in yellow-pink. In the vehicle group, UVA exposure caused fragmentation and degradation of elastin fibers. In contrast, treatment with QC:HPN (8:1) preserved the continuity and organization of elastin fibers, indicating protection against UVA-induced elastin damage. S01–S03 represent skin samples from three independent human donors.

Collagen and Elastin Fiber Production in Human Skin Tissue: The effects of the test product, QC:HPN 8:1, including the formulation on collagen and elastin fiber production in human skin tissue, are summarized in Figure 8, Figure 9 and Figure 10.

Collagen Fiber Production: Figure 8A shows collagen fiber production. UVA exposure significantly reduced collagen fiber production to 78.34 ± 7.77% compared to the negative control (100.00 ± 2.11%, *p* < 0.01), likely due to upregulation of MMP-1, an enzyme that degrades type I and III collagen, consistent with studies showing UVA upregulates MMP-1, which degrades collagen types I and III [18,73]. MMP-1 activation is driven by ROS-mediated NF-κB and AP-1 signaling [18,74].

However, treatment with the test sunscreen restored collagen production to 92.18 ± 2.69%, a statistically significant recovery (*p* < 0.05) versus the UVA-only group, showing a protective effect against UVA-induced collagen degradation. This aligns with previous research showing that topical antioxidants and UV-filters can suppress MMP activity and preserve the dermal matrix. This aligns with research demonstrating that antioxidants like QC suppress MMP-1 activity by scavenging ROS and blocking NF-κB pathways [75].

Elastin Fiber Production: Figure 8B shows elastin fiber production. Similarly, UVA exposure markedly decreased elastin fiber production to 34.19 ± 15.64% (vs. 100.00 ± 14.02% in the control, *p* < 0.001). Elastin degradation, primarily driven by MMP-12 and neutrophil elastase under UVA stress, contributes to skin laxity and wrinkle formation. attributed to MMP-12 and neutrophil elastase activation [73]. Elastase activity is exacerbated by oxidative stress and inflammatory cytokines [12,74]. Application of the test sunscreen significantly increased elastin production to 74.65 ± 16.46% (*p* < 0.05), indicating its potential to mitigate UVA-induced elastin loss, either by inhibiting elastase activity or enhancing elastin synthesis. Polyphenols like QC stabilize elastin fibers by chelating metal ions required for elastase activity [12,75].

Histological Analysis: Masson’s trichrome staining (Figure 9) confirmed these biochemical findings, showing dense and organized collagen fibers in control skin, while UVA-exposed tissue exhibited fragmented and diminished collagen. TS maintained fiber density and organization similar to the control group. Similar findings are reported in studies using antioxidants to preserve dermal structure [18,76].

Verhoeff–Van Gieson (VVG) staining (Figure 10) revealed intact and well-aligned elastin fibers in the control group, which were significantly degraded after UVA exposure. In contrast, TS samples showed preserved elastin morphology, closely resembling the negative control. This mirrors results where polyphenols like EGCG prevent elastin fragmentation by reducing oxidative damage [73,75].

Overall, these results demonstrate that QC:HPN 8:1 formular effectively protects collagen and elastin fibers from UVA-induced damage, both quantitatively and histologically, supporting its role in maintaining dermal structural integrity under UV stress.

## 4. Discussion

In this study, we evaluated the synergistic photoprotective effects of QC and HPN, comparing their efficacy to AGR in models of PLE. While QC shows strong antioxidative and anti-inflammatory effects, its cytotoxicity at concentrations ≥ 10 μg/mL raises safety concerns, particularly for immune-related cells. Nonetheless, concentrations below 10 μg/mL are currently considered safe. Our data reveal that a QC:HPN ratio of 8:1 achieves robust protection against UVA- and blue-light-induced oxidative stress, inflammation, and dermal damage. This effect appears comparable—and in some assays superior—to AGR, positioning this natural flavonoid combination as a promising alternative for PLE management and photoaging prevention. The 8:1 weight ratio of quercetin to hesperidin was selected based on systematic screening of antioxidant enzyme activity, mast cell degranulation, and cytokine suppression under HEV and allergic stimulation conditions. This ratio achieved optimal therapeutic efficacy while maintaining biocompatibility, balancing quercetin’s strong but dose-sensitive bioactivity with hesperidin’s stabilizing, anti-inflammatory properties.

Enhanced Antioxidant Defense: The experimental data reveal HEV exposure decreased fibroblast viability to 82.74%, indicating cytotoxicity under oxidative stress. While a reduction in cell viability alone does not directly prove skin aging or pigmentation, it is consistent with previous studies that associate HEV-induced ROS generation with these skin pathologies [46,77]. To further substantiate this mechanism, we additionally observed decreased antioxidant enzyme activities (SOD and CAT) and signs of ECM degradation, which are key markers of photoaging. Our results show that QC:HPN (8:1) notably restored activities of endogenous antioxidant enzymes—SOD) and CAT—following photo-oxidative insult. This observation aligns with prior evidence of QC’s potent free-radical scavenging abilities, attributed to its polyhydroxylated structure [29,78], and HPN’s complementary antioxidant properties. Additionally, the flavonoid–glycerosome formulation strategy (e.g., nanovesicles) has been shown to enhance skin penetration and augment the antioxidant efficacy of QC in vitro [79]. Our complement of SOD and CAT recovery supports these molecular-level mechanistic insights.

Anti-inflammatory and Anti-allergic Actions: One hallmark of PLE is the release of histamine and cytokines like IL-6 and TNF-α, driving pruritic and inflammatory lesions. In our mast cell degranulation assays, QC:HPN (8:1) significantly inhibited β-hexosaminidase secretion, surpassing AGR’s effect. In tandem, both cytokine measurements and ex vivo skin explant analyses reflected superior downregulation of IL-6 and TNF-α by the QC:HPN treatment.

These anti-inflammatory effects are mechanistically plausible: QC is known to modulate key inflammatory pathways (e.g., NF-κB) and inhibit pro-inflammatory enzyme activity [55,80]. HPN specifically attenuates cytokine release, as demonstrated in both animal and cell culture models [81]. Our findings corroborate these external observations and highlight a potentially broader therapeutic effect beyond antioxidant activity.

Photoprotective and Anti-photoaging Effects: Beyond oxidative stress and inflammation, UVA exposure leads to degradation of dermal extracellular matrix (ECM), manifested as decreased collagen and elastin. Our data indicate that QC:HPN restored collagen and elastin synthesis closer to baseline levels post-UVA. These results are consistent with reports that QC can prevent UVA-induced ROS production, maintain ECM integrity, and reduce markers of photoaging in skin fibroblasts and keratinocytes. Likewise, HPN has shown protective effects in skin models against UV-induced dermal degradation [82].

Comparative Efficacy vs. AGR: AGR is a semi-synthetic flavonoid with recognized UVA-protective activity and proven clinical benefit in PLE [29]. While AGR performed strongly in most assays, the QC:HPN blend equaled or even surpassed its performance in key endpoints: antioxidant enzyme restoration, mast cell stabilization, cytokine suppression, and ECM preservation. These results are noteworthy given AGR’s established role. These findings are consistent with AGR’s known high epidermal bioavailability and potent antioxidant capacity, supporting its strong photoprotective efficacy in skin cells [32]. The synergy between QC and HPN likely reflects complementary pathways—QC as a radical scavenger and HPN as an anti-inflammatory mediator—resulting in a more robust multi-mechanistic protection.

Clinical Implications and Future Directions: Animal and in vitro studies demonstrate that combinations of QC and HPN can synergistically enhance antioxidant capacity. For example, in models of nephrotoxicity, co-administration of QC and HPN significantly reduced oxidative stress markers (MDA, H_2_O_2_, NO) and restored antioxidant enzyme levels (SOD, CAT, GPx, GSH) [58]. This supports the experimental observation that QC:HPN combinations—especially at higher QC ratios—outperform single treatments in restoring enzyme activity.

A topical formulation containing QC:HPN (8:1) may offer a multifunctional strategy for PLE prevention with a lower risk of synthetic sensitization compared to AGR. By targeting key mechanisms of photoaging, it also holds promise for broader skincare applications aligned with the growing demand for natural, clean-label cosmetics. Notably, the QC:HPN 8:1 combination outperformed both its individual components and AGR in restoring antioxidant enzyme activity, inhibiting mast cell degranulation, and suppressing pro-inflammatory cytokines. This synergistic efficacy highlights QC:HPN 8:1 as a novel, multi-targeted therapeutic option not previously reported in PLE or related photoallergic disorders.

While this study demonstrates that the QC:HPN 8:1 complex outperforms AGR in antioxidant activity, cytokine suppression, and mast cell stabilization in vitro and ex vivo, future work is needed to translate these findings into clinical practice. Specifically, formulation studies evaluating skin penetration, photostability, and long-term dermal tolerability under in-use conditions are essential. Additionally, randomized, placebo-controlled clinical trials—including intra-individual or formulation-comparison studies similar to those conducted for AGR-based sunscreens—are required to validate efficacy in patients with PLE, photoallergic dermatitis, or other UV-induced inflammatory dermatoses. Dose and application frequency studies should also be conducted to determine optimal topical concentrations and long-term prophylactic regimens. Although the present study focused on assessing photoprotection using individual compounds, future work should investigate whether the QC:HPN combination demonstrates enhanced photoprotective effects in direct comparison to single agents. This will provide a more comprehensive understanding of its potential clinical application.

To facilitate clinical application, formulation optimization should focus on enhancing the stability and skin penetration of QC and HPN, potentially through advanced delivery systems such as nanovesicles or glycerosomes, which have shown promise in previous research [79]. Safety and irritation assessments remain essential, as both compounds are generally well tolerated but may occasionally cause contact sensitivity.

Given its natural origin, low cytotoxicity, and superior antioxidant, anti-inflammatory, and mast cell-stabilizing properties demonstrated in vitro and ex vivo, QC:HPN 8:1 shows strong potential as a dermatology-focused photoprotective cosmeceutical.

## 5. Conclusions

This study demonstrates that the combination of QC and HPN, particularly at an 8:1 ratio, offers significant photoprotective, anti-inflammatory, and anti-photoaging benefits in cellular and ex vivo models of PLE. Compared to AGR—a synthetic antioxidant widely used in sunscreens—the QC:HPN blend exhibited superior or comparable efficacy in restoring antioxidant enzyme activity, inhibiting mast cell degranulation, reducing inflammatory cytokine production, and preserving dermal collagen and elastin fibers.

Importantly, unlike AGR, which does not occur naturally and requires chemical modification for cosmetic use, the QC:HPN complex is derived from plant-based, naturally occurring compounds. This gives it a critical advantage in terms of biocompatibility, environmental friendliness, and appeal to consumers seeking clean, natural ingredients. Furthermore, safety concerns associated with AGR—such as potential skin sensitization and aquatic toxicity—highlight the need for safer, eco-conscious alternatives.

Taken together, the optimized QC:HPN 8:1 formulation offers a promising natural alternative to AGR for the prevention and management of PLE and UV-induced skin damage. It demonstrates superior protection against oxidative stress and inflammation while maintaining favorable biocompatibility. These findings provide a strong foundation for the future development of topical products targeting PLE and photoaging. Further research should focus on formulation refinement, enhanced dermal delivery, and well-designed clinical trials to enable real-world application and support regulatory approval.

## Data Availability

The data that support the findings of this study are available from the corresponding author upon reasonable request.

## Abbreviations

The following abbreviations are used in this manuscript:

PLE	Polymorphous Light Eruption
QC	Quercetin
HPN	Hesperidin
AGR	Alpha-Glucosylrutin
HEV	High-Energy Visible (light)
ROS	Reactive Oxygen Species
SOD	Superoxide Dismutase
CAT	Catalase
MMP	Matrix Metalloproteinases
NF-κB	Nuclear Factor kappa-light-chain-enhancer of activated B cells
MAPK	Mitogen-Activated Protein Kinase
ELISA	Enzyme-Linked Immunosorbent Assay
